# The diagnostic accuracy of acute phase proteins and proinflammatory cytokines in sheep with pneumonic pasteurellosis

**DOI:** 10.7717/peerj.2161

**Published:** 2016-07-19

**Authors:** Wael M. El-Deeb, Ahmed M. Elmoslemany

**Affiliations:** 1 Department of Clinical Studies, College of Veterinary Medicine and Animal Resources, King Faisal University, Al-Ahsa, Saudi Arabia; 2 Department of Internal Medicine, Infectious Diseases and Fish Diseases, Faculty of Veterinary Medicine, Mansoura University, Mansoura, Egypt; 3 Faculty of Veterinary Medicine, Hygiene and Preventive Medicine, Kafrelsheikh University, Kafrelsheikh, Egypt

**Keywords:** Pneumonic pasteurellosis, Acute phase proteins, Cytokines, Sheep, Diagnostic accuracy, Haptoglobin

## Abstract

The goal of this study was to assess the diagnostic accuracy of acute phase proteins and proinflammatory cytokines in sheep with pneumonic pasteurellosis. Blood samples were collected from 56 sheep (36 naturally infected with * Pasteurella multocida* and 20 healthy controls) belonging to one farm in Eastern region, Saudi Arabia. Serum samples were evaluated for acute phase proteins (Haptoglobin (Hp), serum amyloid A (SAA) and fibrinogen (Fb)), and the proinflammatory cytokines (interleukins (IL-1*α*, IL-1*β*, and IL-6), tumor necrosis factor-alpha (TNF-*α*), and interferon-gamma (IFN-ϒ)). Additionally, nasopharyngeal swabs and bronchoalveolar lavages were collected from all animals for bacteriological examinations. Receiver operating characteristic curve was used to assess the diagnostic performance of each parameter. All parameters showed moderate to high degree of positive correlation with case-control status. There was no significant difference in the area under the curve (AUC) among acute phase proteins; however, both Hp and SAA showed better sensitivity and specificity than Fb. The proinflammatory cytokines (IL1-*α*, IL1-*β*, and IL6) showed similar and highly accurate diagnostic performance (*AUC* > 0.9), whereas IFN-ϒ was moderately accurate (*AUC* = 0.79). In conclusion, this study confirms the value of acute phase proteins and cytokines as diagnostic biomarkers of naturally occuring pneumonic pasteurellosis in sheep.

## Introduction

Pneumonia is an inflammatory response of the alveoli in the lungs to infective agents, resulting in lung consolidation. It is a common disease of sheep in all major sheep-producing countries (Yener et al., 2009). The disease is multifactorial, resulting from dynamic interactions between host, infectious agent and environmental factors. When the bacterial population reach a certain threshold, host susceptibility, non-specific defense mechanisms and lung defense mechanisms become compromised, allowing diseases to occur (Bruere, West & Ridler, 2002).

Pneumonic pasteurellosis and systemic pasteurellosis are the two main diseases caused by pasteurella species in ovine (Donachie, 2000; Rad et al., 2011). Although pasteurellas are opportunistic pathogens which commonly inhabit the upper respiratory tract of ruminants (Dowling et al., 2002; Ackermann & Brogden, 2000; Yener et al., 2009), they become pathogenic when the host is exposed to either stressful environment or infection with primary respiratory pathogens such as viral agents or Mycoplasma spp. Infection with pneumonic pasteurellosis can cause serious economic losses in sheep population resulting from fatalities in acute outbreaks and reduced productivity of chronically infected animals.

Acute phase proteins (APPs) are plasma proteins which increase or decrease in concentration in response to infection, inflammation and internal or external challenges. Monitoring changes in APPs levels have been shown to provide valuable diagnostic and prognostic information during infection and inflammation. However, there are substantial variations in acute phase response between different species (Eckersall, 2000). In small ruminants, some APPs levels change similarly in both sheep and goat, whereas other APPs show different magnitude of response between the two species (Gonzalez et al., 2008).

In bovine and camels, several studies have been conducted to elucidate the role of APPs in different disorders including parasitic and bacterial infection (El-Deeb & Iacob, 2012; El-Deeb, Fouda & El-Bahr, 2014; El-Deeb, 2015; El-Deeb & Elmoslemany, 2016; El-Deeb & Buczinski, 2015). Conversely, only a limited number of studies have been done regarding specific bacterial, viral or parasitic infections in ovine species (El-Deeb, 2013; El-Deeb & Tharwat, 2015). Furthermore, these studies focused mainly on changes in the level of APPs but provided little information on their diagnostic accuracy. Therefore, the goal of this study was to assess the diagnostic accuracy of APPs and inflammatory cytokines in sheep infected with pneumonic pasteurellosis.

## Materials and Methods

### Study animals

This study was conducted initially on 73 Naimi sheep, three to four years old (53 cases and 20 healthy control) belonging to a flock of free grazing sheep (*n* = 543) in Al-Ahsa region, Saudi Arabia. The project was approved by the animal care committee at King Faisal University (number 130031). The flock was examined following reports of respiratory disease problems. All sheep were clinically examined with direct observation and recording of clinical signs. Additionally, nasopharyngeal swabs and bronchoalveolar lavages were collected from sheep exhibiting signs of clinical disease for bacteriologic examination. Sheep were classified into cases and controls based on clinical signs, culture results and autopsy for dead animals. Cases were defined as sheep with clinical sings of pneumonia and positive culture for *Pasteurella multocida (p. multocida)*. Healthy controls were sheep with no clinical signs and negative culture results. Seven animals (with positive nasal swabs, negative bronchoalveolar lavages, and with no clinical signs of pneumonia) and ten animals (with other bacterial infections) were excluded from the study to avoid misclassification of control sheep as being cases. Finally, only 36 sheep that satisfied case definition and 20 healthy controls (randomly selected from healthy sheep) were considered for further analysis in this study.

### Sampling

Serum samples were collected from all 56 sheep and stored at −20 °C until biochemical analyses were performed. Moreover, nasopharyngeal swabs and bronchoalveolar lavage were collected from all animals under investigation (*N* = 73). Finally, heart-blood and lung samples (from necropsied sheep (*n* = 6)) which died after initial serum sampling were also collected for bacteriological examinations.

### Determination of acute phase proteins and inflammatory cytokines

Hp levels were detected in serum samples via preservation of the peroxidase activity of haemoglobin, which is directly proportional to the amount of Hp (Tridelta Development Plc.). SAA was measured in serum samples by a solid phase sandwich ELISA (Tridelta Development Plc.).

The levels of inflammatory cytokines IL-6, TNF-*α*, IL-1*α*, IL-1*β*, and IFN-ϒ were measured from serum samples using commercially available sheep ELISA Kits (CUSABIO).

### Isolation and identification of *P. multocida*

All samples were cultured for isolation and identification of *P. multocida* based on cultural, morphological and biochemical characteristics using standard bacteriological techniques (Carter & Cole Jr, 1990).

### Statistical analysis

Descriptive statistics were calculated as mean, median, and 25th and 75th percentiles for each parameter by disease status (pneumonic cases vs. controls). The mean ranks for each parameter were compared between cases and control groups using non-parametric Wilcoxon rank-sum (Mann–Whitney) test due to significant deviation of the data from normality and failure to normalize data with transformation. Differences between cases and control were considered statistically significant at *P* < 0.05. Correlation among different parameters was assessed using Spearman’s correlation coefficient.

**Figure 1:**
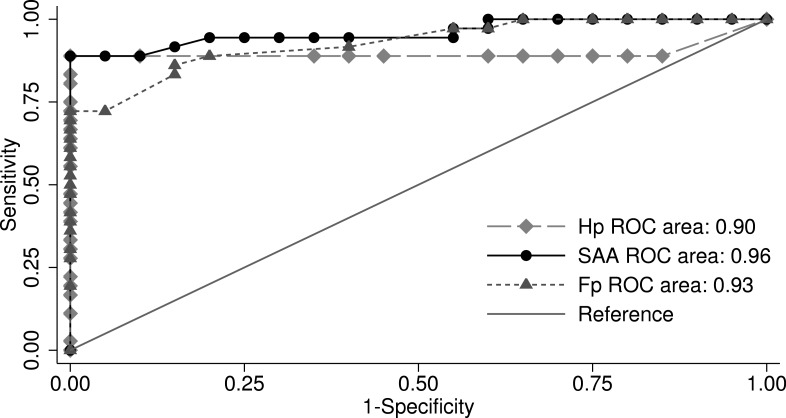
Receiver operating characteristic plot: comparison of the area under the curve (AUC) for haptoglobin (Hp), serum amyloid A (SAA), and fibrinogen (Fb).

Selection of cut-off points that optimize sensitivity and specificity for each parameter were determined using non-parametric receiver operating characteristic curve (ROC). The ROC curves were constructed by plotting 1-specificity (*x*-axis) versus sensitivity (*y*-axis) for all possible threshold values of the parameter to be evaluated. The area under the curve (AUC) indicates the overall accuracy of the tested parameter. A biomarker with no predictive value would have an AUC of 0.5 (represented by the diagonal line in the ROC plot Fig. 1), while a biomarker with perfect ability to predict disease would have an AUC of one. Values of AUC between 0.5 and one are interpreted as low (0.5 > AUC ≤ 0.7), moderate (0.7 > AUC ≤ 0.9), or high (0.9 > AUC < 1), accuracy (Swets, 1988). The two-graph ROC (TGROC) plot was also used to graph variation of sensitivity and specificity of biomarker across a range of cut-offs. All analyses were done using Stata version 13 (Stata Corp).

## Results

Table 1 shows significantly higher levels of Hp, SAA, and Fb in cases compared to controls, though the increase was more pronounced (several folds) in both Hp and SAA levels. Additionally, a significant increase (*P* < 0.05) in the level of proinflammatory cytokines was also observed in the cases.

**Table 1:** Summary statistics of the level of acute phase proteins (APP) and proinflammatory cytokines in control and pneumonic sheep.

Parameter	Control (*N* = 20)				Cases (*N* = 36)				
	Mean	Median	25%	75%	Mean	Median	25%	75%	*P*^a^ value
Hp (g/L)	0.048	0.054	0.047	0.062	1.65	1.87	1.65	2.14	<0.0001
SAA (µg/mL)	4.32	4.56	3.95	4.87	26.89	29.36	28.26	30.75	<0.0001
Fb (g/L)	2.34	2.36	2.27	2.41	3.47	3.65	2.51	4.12	<0.0001
IL1-*α* (pg/ml)	13.48	13.75	12.36	14.74	24.75	26.35	24.96	27.81	<0.0001
IL1-*β* (pg/ml)	18.49	18.52	17.26	19.45	29.30	30.35	30.12	31.80	<0.0001
IL6 (pg/ml)	10.63	10.40	9.51	11.40	16.27	17.25	15.47	18.24	<0.0001
TNF-*α* (pg/ml)	8.63	8.57	8.05	9.25	17.57	19.25	18.31	19.84	<0.0001
IFN-*γ* (pg/ml)	10.02	10.31	9.07	10.79	14.53	16.30	10.36	17.25	<0.0001

**Notes.**

a *P* value resulting from non-parametric Wilcoxon Mann–Whitney test.

Table 2 shows correlation coefficients (*r*) among study parameters. All measured biomarkers had a significant (*P* < 0.05) positive correlation with pneumonic cases and most of the studied biomarkers were moderately correlated. SAA showed the highest correlation with pneumonic cases (*r* = 0.74). A high correlation (*r* ≥ 0.75) was observed between Hp & IL1-*α*, and also among different interleukins. A low correlation (*r* ≤ 0.34) was observed between IFN-*γ* and most of the other parameters.

**Table 2:** Correlation matrix among acute phase proteins (APPs) and proinflammatory cytokines in 56 sheep (20 control and 36 with pneumonic pasteurellosis).

Parameter	Case control	Hp	SAA	Fb	IL1-*α*	IL1-*β*	IL6	TNF-*α*
Hp	0.66							
SAA	0.74	0.51	1.00					
Fb	0.71	0.53	0.61	1.00				
IL1-*α*	0.68	0.75	0.38	0.48	1.00			
IL1-*β*	0.70	0.71	0.47	0.50	0.71	1.00		
IL6	0.68	0.73	0.42	0.51	0.78	0.77	1.00	
TNF-*α*	0.72	0.50	0.54	0.56	0.58	0.49	0.51	1.00
IFN-*γ*	0.48	0.33	0.33	0.48	0.25	0.31	0.25	0.34

**Figure 2:**
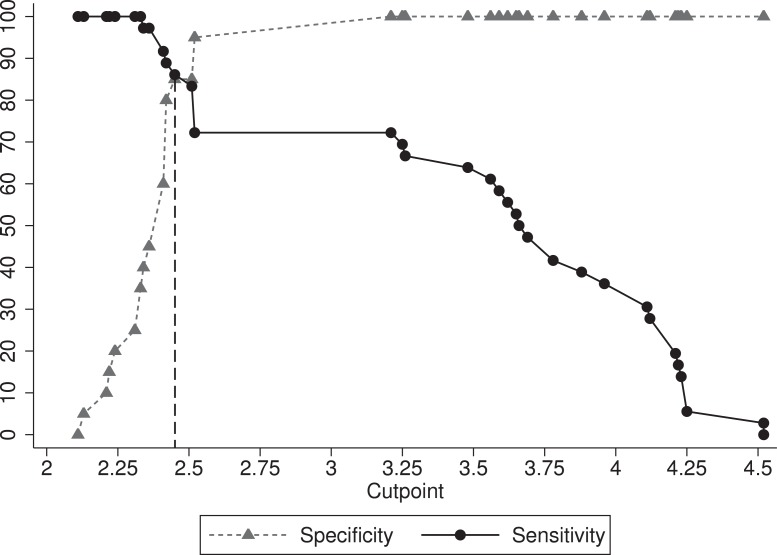
Two-graph receiver operating characteristic (TG-ROC) plot showing optimal cut-off and variation in sensitivity and specificity over various cut-off points of fibrinogen (Fb).

The results of the ROC analysis for evaluation of the overall diagnostic accuracy of APPs and cytokines are shown in Table 3. Comparison of the AUC indicated no significant difference (*P* = 0.49) in the AUC among Hp (0.90), SAA (0.96), and Fb (0.93) (Fig. 1). However, both Hp and SAA showed better sensitivity and specificity than Fb at selected cut-offs (Table 3). Using ROC analysis and TGROC, the optimum cut-offs for discrimination between pneumonic and control sheep for Hp, SAA and Fb were 0.065, 5.26 and 2.45 g/L, respectively (Table 3). Figure 2 illustrates the optimal cut-off and variation in sensitivity and specificity of Fb at different cut-off points.

**Table 3:** Test characteristics of acute phase proteins (APPs) and proinflammatory cytokines for diagnosis of pneumonic pasteurellosis in sheep.

Parameter	Threshold	Sensitivity (%)	Specificity (%)	% correctly classified	AUC (95% CI)^a^
Hp (g/L)	≥0.065	89	100	93	0.90 (0.78–0.96)
SAA (µg/mL)	≥5.26	89	95	91	0.96 (0.88–0.99)
Fb (g/L)	≥2.45	86	85	86	0.93 (0.83–0.98)
IL1-*α* (pg/ml)	≥16.25	86	95	89	0.91 (0.80–0.97)
IL1-*β* (pg/ml)	≥29	86	100	91	0.92 (0.85–0.99)
IL6 (pg/ml)	≥15	86	100	91	0.91 (0.83–0.99)
TNF-*α* (pg/ml)	≥17	83	100	89	0.93 (0.87–1.00)
IFN-*γ* (pg/ml)	≥15	69	100	80	0.79 (0.67–0.91)

**Notes.**

a 95% CI, 95% confidence interval.

The proinflammatory cytokines IL1-*α*, IL1-*β*, and IL6 showed similar and highly accurate diagnostic performance (*AUC* > 0.9), whereas IFN-ϒ was moderately accurate in differentiating between pneumonic and control sheep (AUC = 0.79) (Table 3).

## Discussion

To the best of our knowledge, our data is the first to explore the diagnostic accuracy of APPs and inflammatory cytokines in sheep with pneumonic pasteurellosis under natural field condition.

Overall, there is limited information on the relationship between APPs and naturally occurring pneumonic pasteurellosis in sheep. Hp acts as a major APP in ruminants, it could be detected in subclinically diseased animals and its serum level is used as a biological marker of disease severity (Godson et al., 1996). In our study, Hp concentration increased 34 times in pneumonic sheep indicating a major response to the infection. The increased plasma level of Hp could be induced by tissue damage resulting from infection or inflammation (Blackmore, 1988). Hp has bacteriostatic effects by binding free haemoglobin, thus depriving bacteria from iron required for their growth (Eaton et al., 1982). Our results are consistent with previous studies on calves with respiratory diseases due to natural (Humblet et al., 2004; Nikunen et al., 2007) or experimental (Dowling et al., 2002) infection with *P. multocida*. Similar results were also observed in sheep with experimentally induced pneumonia (Pfeffer & Rogers, 1989) and buffalo calves with bacterial bronchopneumonia (El-Deeb, 2011; El-Bahr & El-Deeb, 2013). In contrast, some studies demonstrated limited association between Hp and respiratory diseases in feedlot cattle (Wittum et al., 1996; Young et al., 1996). This study also showed that SAA level increased approximately 7 folds in pneumonic sheep compared to healthy one indicating a moderate response, this could be attributed to the physiological role of SAA in host defense during inflammation (Murata, Shimada & Yoshioka, 2004; Orro et al., 2011; Urieli-Shoval, Linke & Matzner, 2000). SAA has a role in alteration of cholesterol metabolism under inflammatory conditions (Pannen & Robotham, 1995). Moreover, SAA binds Gram-negative bacteria (Hari-Dass et al., 2005), possibly to facilitate the uptake by macrophages and neutrophils (Larson et al., 2005). Similarly, marked elevation of SAA was reported in pneumonic calves (Horadagoda et al., 1994; Nikunen et al., 2007; Orro et al., 2011). Although, some studies suggested that SAA is a more sensitive biomarker for viral infections (Heegaard et al., 2000) and acute inflammation (Horadagoda et al., 1994). Others indicated that Hp might be preferred to SAA in detecting respiratory disease in calves under field conditions (Angen et al., 2009). In the former study, SAA showed more rapid response, whereas Hp was more correlated with severity of clinical signs and degree of lung consolidation (Heegaard et al., 2000). Fb is classified as a minor APP, which is characterized by a minor response with a maximum increase of about twice the normal concentrations during infection or inflammation (Hirvonen, Hietakorpi & Saloniemi, 1997). Fb is also considered a consistent marker of bacterial infection and inflammation in domestic ruminants (Gonzalez et al., 2008; Nikunen et al., 2007; Pfeffer et al., 1993; Youssef, El-khodery & Abdo, 2015). In our study, the concentration of serum Fb in pneumonic sheep was approximately twice that of the control group. This elevation may be attributed to the involvement of Fb in modulating hemostasis, inflammatory response, and the tissue repairing process (Feldman et al., 2000).

The different types of inflammatory cytokines are the principal stimulators of APPs gene expression, and each kind of cytokines recruits a different type of APPs (Baumann & Gauldie, 1994). Thus, the elevated levels of APPs seen in this study reveal the secretion of different amounts or types of inflammatory cytokines. Comparable results regarding such high levels of IL1-*β*, TNF-*α* and IFN-*γ* were detected in pigs with viral respiratory disease (Van Reeth & Nauwynck, 2000) and cattle with bacterial infection (Pace et al., 1993; Horadagoda et al., 1994; Morsey et al., 1999; Kasimanickam et al., 2013; El-Deeb & Elmoslemany, 2016). In addition, the expression of IL1-*β* and TNF-*α* were elevated in the respiratory airways and lung lesions of diseased calves with pneumonic pasteurellosis (Malazdrewich et al., 2001).

The ability of APPs to discriminate between pneumonic and healthy sheep was evaluated using ROC analysis. Under the conditions of this study, all APPs showed a high degree of discrimination between pneumonic and control sheep (*AUC* ≥ 0.9) according to guidelines reported by Swets (1988). However, both Hp and SAA showed better sensitivity and specificity than Fb resulting in better overall correct classification of pneumonic and control sheep. Selection of cut-off point for each parameters was based on optimizing sensitivity and specificity which lead to the best overall correct classification. The ability of Hp and SAA to discriminate between control and clinical cases may be related to the large difference in their concentration between control and clinical cases. The proinflammatory cytokines IL1-*α*, IL1-*β*, and IL6 showed similar and highly accurate diagnostic performance (*AUC* > 0.9), whereas IFN-*γ* was moderately accurate in differentiation between control and pneumonic sheep (AUC = 0.79). Our results also indicate a high diagnostic accuracy of the measured cytokines. However, APPs may serve as better biomarkers of inflammation considering the very short half-life of cytokines.

## Conclusions

The results of this study indicate that pneumonic pasteurellosis caused by *P. multocida* in sheep was associated with a significant increase in APPs and cytokines, with the greatest increase being observed in Hp. There was a moderate to high correlation between disease status and APPs and cytokines concentrations. Finally, this study highlights the value of acute phase proteins and cytokines as diagnostic biomarkers of naturally occuring pneumonic pasteurellosis in sheep.

##  Supplemental Information

10.7717/peerj.2161/supp-1 Data S1 Raw data

## Additional Information and Declarations

### Data Availability

The following information was supplied regarding data availability: The raw data has been supplied as Data S1.
